# Impact of clinical frailty on surgical and non-surgical complications after major emergency abdominal surgery

**DOI:** 10.1093/bjsopen/zrae039

**Published:** 2024-05-24

**Authors:** Christian Snitkjær, Lasse Rehné Jensen, Liv í Soylu, Camilla Hauge, Madeline Kvist, Thomas K Jensen, Dunja Kokotovic, Jakob Burcharth

**Affiliations:** Department of Gastrointestinal and Hepatic Diseases, Copenhagen University Hospital—Herlev and Gentofte, Herlev, Denmark; Emergency Surgery Research Group Copenhagen (EMERGE Cph), Copenhagen University Hospital—Herlev and Gentofte, Herlev, Denmark; Department of Gastrointestinal and Hepatic Diseases, Copenhagen University Hospital—Herlev and Gentofte, Herlev, Denmark; Emergency Surgery Research Group Copenhagen (EMERGE Cph), Copenhagen University Hospital—Herlev and Gentofte, Herlev, Denmark; Department of Gastrointestinal and Hepatic Diseases, Copenhagen University Hospital—Herlev and Gentofte, Herlev, Denmark; Emergency Surgery Research Group Copenhagen (EMERGE Cph), Copenhagen University Hospital—Herlev and Gentofte, Herlev, Denmark; Department of Gastrointestinal and Hepatic Diseases, Copenhagen University Hospital—Herlev and Gentofte, Herlev, Denmark; Emergency Surgery Research Group Copenhagen (EMERGE Cph), Copenhagen University Hospital—Herlev and Gentofte, Herlev, Denmark; Department of Gastrointestinal and Hepatic Diseases, Copenhagen University Hospital—Herlev and Gentofte, Herlev, Denmark; Emergency Surgery Research Group Copenhagen (EMERGE Cph), Copenhagen University Hospital—Herlev and Gentofte, Herlev, Denmark; Department of Gastrointestinal and Hepatic Diseases, Copenhagen University Hospital—Herlev and Gentofte, Herlev, Denmark; Emergency Surgery Research Group Copenhagen (EMERGE Cph), Copenhagen University Hospital—Herlev and Gentofte, Herlev, Denmark; Department of Gastrointestinal and Hepatic Diseases, Copenhagen University Hospital—Herlev and Gentofte, Herlev, Denmark; Emergency Surgery Research Group Copenhagen (EMERGE Cph), Copenhagen University Hospital—Herlev and Gentofte, Herlev, Denmark; Department of Gastrointestinal and Hepatic Diseases, Copenhagen University Hospital—Herlev and Gentofte, Herlev, Denmark; Emergency Surgery Research Group Copenhagen (EMERGE Cph), Copenhagen University Hospital—Herlev and Gentofte, Herlev, Denmark

## Abstract

**Background:**

Major emergency abdominal surgery is associated with a high risk of morbidity and mortality. Given the ageing and increasingly frail population, understanding the impact of frailty on complication patterns after surgery is crucial. The aim of this study was to evaluate the association between clinical frailty and organ-specific postoperative complications after major emergency abdominal surgery.

**Methods:**

A prospective cohort study including all patients undergoing major emergency abdominal surgery at Copenhagen University Hospital Herlev, Denmark, from 1 October 2020 to 1 August 2022, was performed. Clinical frailty scale scores were determined for all patients upon admission and patients were then analysed according to clinical frailty scale groups (scores of 1–3, 4–6, or 7–9). Postoperative complications were registered until discharge.

**Results:**

A total of 520 patients were identified. Patients with a low clinical frailty scale score (1–3) experienced fewer total complications (120 complications per 100 patients) compared with patients with clinical frailty scale scores of 4–6 (250 complications per 100 patients) and 7–9 (277 complications per 100 patients) (*P* < 0.001). A high clinical frailty scale score was associated with a high risk of pneumonia (*P* = 0.009), delirium (*P* < 0.001), atrial fibrillation (*P* = 0.020), and infectious complications in general (*P* < 0.001). Patients with severe frailty (clinical frailty scale score of 7–9) suffered from more surgical complications (*P* = 0.001) compared with the rest of the cohort. Severe frailty was associated with a high risk of 30-day mortality (33% for patients with a clinical frailty scale score of 7–9 *versus* 3.6% for patients with a clinical frailty scale score of 1–3, *P* < 0.001). In a multivariate analysis, an increasing degree of clinical frailty was found to be significantly associated with developing at least one complication.

**Conclusion:**

Patients with frailty have a significantly increased risk of postoperative complications after major emergency abdominal surgery, especially atrial fibrillation, delirium, and pneumonia. Likewise, patients with frailty have an increased risk of mortality within 90 days. Thus, frailty is a significant predictor for adverse events after major emergency abdominal surgery and should be considered in all patients undergoing major emergency abdominal surgery.

## Teaser

Patients with any degree of clinical frailty have a significantly increased risk of developing postoperative complications, notably medical complications, such as pneumonia, atrial fibrillation, delirium, and sepsis, compared with patients without frailty. Furthermore, patients with clinical frailty have a significantly increased risk of short-term mortality compared with patients without frailty.

## Introduction

Major emergency abdominal surgery (MEAS) is a surgical procedure that is performed urgently to address potential life-threatening intra-abdominal conditions, such as small or large bowel obstruction, perforated peptic ulcer, bowel perforation, intestinal ischaemia, anastomotic leakage, and severe intra-abdominal bleeding. These conditions pose a significant challenge due to the high risk of morbidity and both short- and long-term mortality^1–4^. Furthermore, with the global population increasing and life expectancy rising, an increasing number of elderly and frail patients are undergoing MEAS^5^.

Frailty is a complex state that arises from age- and disease-related deficiencies, resulting in decreased physical capacity and heightened vulnerability to internal and external stressors^6^. Sarcopenia significantly predicts clinical frailty^7–9^. The concept of frailty lends itself to evaluation through diverse scoring systems, including the clinical frailty scale (CFS)^9^. Increased frailty has been linked to an increased risk of short-term mortality and an increased risk of postoperative complications as a whole^10–12^. In elective surgery, frailty has been associated with an elevated risk of postoperative complications, prolonged hospital stay, long-term mortality, and ICU admission^13–18^. In the context of MEAS, the current literature on the topic remains scarce^19,20^, with the few existing studies focusing on the association between frailty and short-term mortality^21–23^.

The authors hypothesized that frailty would associate with an organ-specific pattern of complications regarding surgical and non-surgical complications. The aim of this study was to evaluate the in-hospital complications encountered by patients undergoing MEAS and their potential association with frailty.

## Methods

### Study design and setting

A prospective cohort study was conducted, including patients undergoing MEAS between 1 October 2020 and 1 August 2022 at Copenhagen University Hospital Herlev, Denmark. Copenhagen University Hospital Herlev has an emergency facility serving approximately 450 000 individuals. The department has adopted a perioperative care bundle that covers all aspects for patients with severe intra-abdominal conditions who require MEAS. This framework, known in Denmark as Acute High-risk Abdominal surgery (AHA)^24^, consists, before surgery, of a standardized protocol that features rapid clinical assessment, acute CT scan within 2 h, intravenous antibiotics upon suspicion of an AHA diagnosis, nasogastric tube insertion, initial fluid therapy, epidural anaesthesia unless contraindicated, and expedited surgical intervention when warranted^25^. Intraoperatively, the department has specific instructions, depending on the indication and physiological condition^26^, and, after surgery, patients are triaged to a high-dependency unit, with subsequent admission to a dedicated AHA ward that is covered exclusively by consultant emergency surgeons, with standardized protocols for observation, pain management, mobilization, nutrition, and discharge^27^.

Clinical frailty was identified in the included cohort before surgery by assessing baseline functional level using the CFS^9^. The CFS was selected as the preferred measure of frailty due to its suitability for clinical application and relevance in assessing patients’ overall health and functional status. The CFS is a numerical assessment tool that quantifies an individual’s baseline available capacity on a scale ranging from 1 to 9. This scale categorizes individuals into three distinct groups: scores of 1–3 indicate no frailty; scores of 4–6 indicate mild to moderate frailty; and scores of 7–9 indicate severe frailty, including patients who most likely will not survive only minor disease (CFS 8) and terminally ill patients with an expected life expectancy of less than 6 months (CFS 9). Thus, frailty, in this study, was defined as a CFS score of 4–9, with subgroups of mild to moderate frailty and severe frailty, as mentioned above.

### Inclusion and exclusion

All patients 18 years or older were enrolled in the perioperative care bundle (AHA). Patients who underwent a primary operation or reoperation (for example reoperation after non-MEAS surgery) were included. Patients who did not enrol in the perioperative care bundle (AHA) immediately and did not undergo urgent surgery (defined as within 12 h) were excluded from this analysis.

### Data management

Study data were collected and managed using research electronic data capture (REDCap) tools hosted at the Department of Gastrointestinal and Hepatic Diseases, Copenhagen University Hospital—Herlev and Gentofte, Denmark. REDCap is a secure, web-based software platform designed to support data capture for research studies, providing: an intuitive interface for validated data capture; audit trails for tracking data manipulation and export procedures; automated export procedures for seamless data downloads to common statistical packages; and procedures for data integration and interoperability with external sources^28,29^.

Several clinical variables were included in the data collection, such as baseline data (age, sex, BMI, and ASA grade), preoperative and intraoperative details (such as medication use, previous abdominal surgery, duration between clinical assessment and CT scan, and perioperative findings and strategies), postoperative complications (defined by organ-specific location), and risk scores (such as the CFS score). The study was approved by the Capital Region of Demark (P-2020-1166, R-21038079) and the Danish Data Protection Agency (P-2021-431), did not require ethics approval, in accordance with Danish law, and was conducted and reported in accordance with the STROBE checklist^30^.

### Outcomes

The primary outcome was the organ-specific pattern of postoperative complications (classified into surgical and non-surgical) after MEAS, stratified by CFS score, and the secondary outcome was short-term mortality after MEAS, stratified by CFS score.

### Statistical analysis

All statistical tests were two-sided and *P* < 0.050 was considered statistically significant. The *P* values were computed between two groups: the CFS 1–3 group and the CFS 4–6 group. The objective was to discern the dissimilarities between non-frail individuals and those exhibiting mild to moderate frailty, under the hypothesis that any significant variance observed between CFS 1–3 and CFS 4–6 would potentially indicate detectable differences when compared with the more severe frailty category, CFS 7–9.

Survival analysis was conducted using survival curves and mortality rates to assess the impact of frailty on intrahospital mortality. The log rank test was employed to compare survival curves between the frail and non-frail patient groups. Multivariate regression analysis was performed to identify independent predictors of postoperative complications and mortality. This analysis considered various well-known risk factors of postoperative morbidity, including frailty, laparotomy, and peritonitis, as potential predictors. Adjustments were made for other confounding variables to determine the independent effect of frailty on outcomes. Variables for the multivariate analysis were chosen based on clinical relevance.

The variable BMI was classified into two groups: 18.5–25 kg/m^2^ (reference) and greater than 25 kg/m^2^. Similarly, the variable ASA grade was classified into two groups: I–II (reference) and III–IV. The degree of contamination was classified according to the surgical wound classification (SWC) system: 1, clean; 2, clean contaminated; 3, contaminated; and 4, dirty^31^. The variable SWC was then subdivided into two groups: 1–2 (reference) and 3–4. The surgical approach was classified into two groups: laparoscopy only (reference) and laparoscopy converted to laparotomy and laparotomy only. Age was classified into four groups: less than 60 years; 60–69 years; 70–79 years; and greater than or equal to 80 years.

## Results

### Demographics

**Fig. 1:**
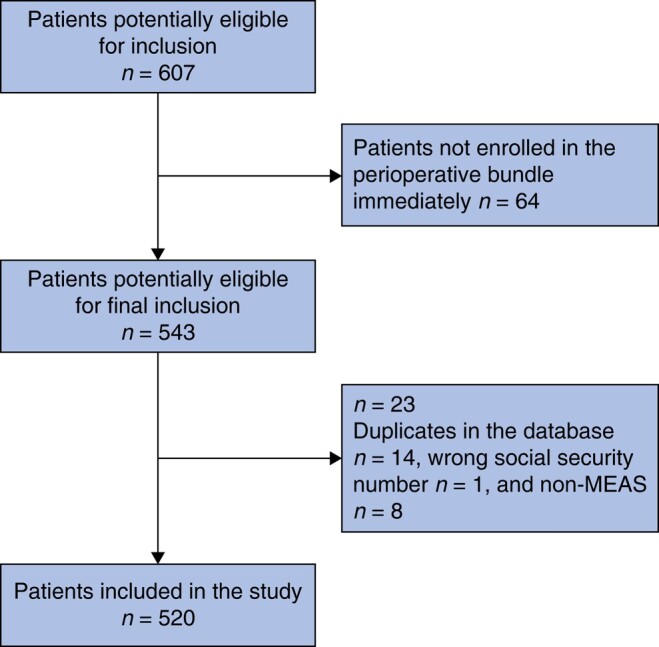
Flow chart showing the inclusion and exclusion of patients in the present study MEAS, major emergency abdominal surgery.

A total of 607 patients were eligible for inclusion. Of these 607 patients, 87 patients were excluded, meaning that a total of 520 patients were included in this study (*Fig. 1*). Then, three categories based on CFS were applied: patients with no frailty (CFS 1–3, 336 patients), patients with mild to moderate frailty (CFS 4–6, 136 patients), and patients with severe frailty (CFS 7–9, 48 patients). For patients in the CFS 7–9 group, 25, 19, and 4 patients were noted for CFS 7, CFS 8, and CFS 9 respectively. Group allocation and baseline data are shown in *Table 1*.

**Table 1:** Baseline data of patients undergoing major emergency abdominal surgery according to the clinical frailty scale

	CFS 1–3, *n* = 336	CFS 4–6, *n* = 136	CFS 7–9, *n* = 48	*P*
Age (years), median (i.q.r.)	62 (50–76)	72 (63–82)	79 (75–89)	0.020*
**Sex**				
Male	160 (47.5)	66 (48.5)	18 (37.5)	0.600
Female	176 (52.5)	70 (51.5)	30 (62.5)	
BMI (kg/m^2^), median (i.q.r.)	24.8 (21.1–27.8)	24.9 (20.9–27.8)	22.9 (19.4–25.0)	0.610
**ASA grade**				
I–II	243 (72.3)	45 (33.1)	7 (15)	<0.001*
III–IV	58 (17.3)	91 (66.9)	41 (85)	0.010*
NA	35 (10.4)	0 (0.0)	0 (0.0)	n.a.
**WHO PS**				
0–1	279 (83.0)	68 (50.0)	7 (15)	0.002*
2–4	20 (6.5)	68 (50.0)	41 (85)	<0.001*
NA	35 (10.5)	0 (0.0)	0 (0.0)	n.a.
**Alcohol consumption per week (units)**				
0	87 (25.9)	33 (24.3)	11 (23)	0.710
1–7	125 (37.2)	57 (41.9)	20 (42)	0.340
8–14	98 (29.2)	28 (20.5)	10 (21)	0.060
>14	26 (7.7)	18 (13.2)	7 (15)	0.060
**Smoking status**				
Never	175 (52.0)	42 (30.9)	12 (25)	<0.001*
Previously (>8 weeks)	112 (33.5)	66 (48.5)	21 (44)	0.002*
Active	49 (14.5)	28 (20.6)	15 (31)	0.110
**Cardiac**	108 (32.1)	97 (71.3)	33 (69)	<0.001*
IHD	22 (6.5)	17 (12.5)	9 (19)	0.030*
Heart failure	7 (2.1)	11 (8.1)	2 (4)	0.002*
Hypertension	71 (21.1)	74 (54.4)	20 (42)	<0.001*
Atrial fibrillation	23 (6.8)	23 (16.9)	10 (21)	<0.001*
Other (valve disease, previous PE or DVT, high-degree AV block)	11 (3.3)	9 (6.6)	7 (15)	0.100
**Endocrinological**	47 (14.0)	35 (25.7)	13 (27)	0.002*
Diabetes type 1 and type 2	18 (5.4)	17 (12.5)	7 (15)	0.007*
Thyroid disease	19 (5.7)	13 (9.6)	2 (4)	0.120
Other (pituitary gland disease, osteoporosis)	10 (3.0)	9 (6.6)	4 (8)	0.070
**Pulmonary**	24 (7.1)	30 (22.1)	9 (19)	<0.001*
COPD	12 (3.6)	19 (14.0)	8 (17)	<0.001*
Asthma	12 (3.6)	10 (7.4)	1 (2)	0.070
Other (lung fibrosis, sarcoidosis)	0 (0.0)	1 (0.7)	0 (0)	n.a.
**Renal**	10 (3.0)	17 (12.5)	4 (8)	<0.001*
CKD	5 (1.5)	13 (9.6)	1 (2)	<0.001*
Hydronephrosis	1 (0.3)	3 (2.2)	1 (2)	n.a.
Other (kidney stone, SLE, pyelonephritis)	4 (1.2)	3 (2.2)	2 (4)	n.a.
**Cerebral**	21 (6.3)	28 (20.6)	19 (40)	<0.001*
Previous stroke or TIA	12 (3.6)	15 (11.0)	11 (23)	<0.001*
Dementia	0 (0.0)	3 (2.2)	5 (10)	n.a.
Other	8 (2.4)	14 (10.3)	5 (10)	0.010*
**Gastroenterological**	26 (7.7)	32 (23.5)	7 (15)	<0.001*
Liver cirrhosis	2 (0.6)	11 (8.1)	2 (4)	<0.001*
IBD	12 (3.6)	4 (2.9)	0 (0)	0.730
Chronic pancreatitis	6 (1.8)	12 (8.8)	5 (10)	<0.001*
Other (steatosis, IBS, microscopic colitis)	5 (1.5)	5 (3.7)	0 (0)	0.010*
**Cancer**				
Never	272 (81.0)	91 (66.9)	31 (65)	0.120
Active cancer	26 (7.7)	21 (15.4)	7 (15)	0.010*
Previous cancer	38 (11.3)	24 (17.6)	10 (21)	0.060
**Previous abdominal surgery**	153 (45.5)	65 (47.8)	13 (27)	0.650
Laparoscopic	86 (25.6)	29 (21.3)	5 (10)	0.330
Open (laparotomy)	67 (19.9)	36 (26.5)	8 (17)	0.120

Values are *n* (%) unless otherwise indicated. *Statistically significant. CFS, clinical frailty scale; i.q.r., interquartile range; PS, performance score; IHD, ischaemic heart disease; PE, pulmonary embolism; DVT, deep-vein thrombosis; AV, atrioventricular block; COPD, chronic obstructive pulmonary disease; CKD, chronic kidney disease; SLE, systemic lupus erythematosus; TIA, transient ischaemic attack; IBD, inflammatory bowel disease; IBS, irritable bowel syndrome.

### Intraoperative data

In total, 292 primary operations and 44 reoperations were identified for patients in the CFS 1–3 group, whereas 122 primary operations and 14 reoperations were identified for patients in the CFS 4–6 group and 47 primary operations and 1 reoperation were identified for patients in the CFS 7–9 group (*P* = 0.400). There were some differences regarding perioperative findings in the different CFS groups. The likelihood of perforation as a perioperative finding increased in frail patients (rates of 22.6%, 33.8%, and 40% for patients in the CFS 1–3, CFS 4–6, and CFS 7–9 groups respectively, *P* = 0.010) and intraoperative malignant findings were more frequently seen in frail patients (rates of 7.1%, 16.9%, and 17% for patients in the CFS 1–3, CFS 4–6, and CFS 7–9 groups respectively, *P* = 0.001; *Table 2*). Patients in the CFS 4–6 and CFS 7–9 groups were more likely to be converted from laparoscopy to laparotomy (rates of 36.0%, 65.4%, and 54% for patients in the CFS 1–3, CFS 4–6, and CFS 7–9 groups respectively, *P* < 0.001).

**Table 2:** Distribution of intraoperative data according to the clinical frailty scale

	CFS 1–3, *n* = 336	CFS 4–6, *n* = 136	CFS 7–9, *n* = 48	*P*
Primary operation	292 (86.9)	122 (89.7)	47 (98)	0.400
Reoperation	44 (13.1)	14 (10.3)	1 (2)	0.400
**Perioperative findings**				
Bowel obstruction	180 (53.6)	71 (52.2)	27 (56)	0.790
SBO	150 (44.6)	55 (40.4)	17 (35)	0.400
LBO	24 (7.1)	8 (5.9)	8 (17)	0.650
Both SBO and LBO	6 (1.8)	8 (5.9)	2 (4)	0.020*
Perforation	76 (22.6)	46 (33.8)	19 (40)	0.010*
Gastric ulcer	6 (1.8)	6 (4.4)	6 (13)	0.100
Duodenal ulcer	6 (1.8)	7 (5.1)	3 (6)	0.040*
Small intestine	24 (7.1)	8 (5.9)	3 (6)	0.620
Large intestine	38 (11.3)	23 (16.9)	6 (13)	0.100
Rectum	0 (0.0)	1 (0.7)	0 (0.0)	n.a.
Anastomotic leakage	4 (1.2)	1 (0.7)	0 (0.0)	n.a.
Degree of peritonitis†				
1	77 (22.9)	8 (5.9)	4 (8)	<0.001*
2	191 (56.8)	86 (63.2)	24 (50)	0.200
3	31 (9.2)	19 (14.0)	8 (17)	0.130
4	37 (11.0)	23 (16.9)	12 (25)	0.080
No	328 (97.6)	331 (98.5)	46 (96)	0.400
Intraoperative malignant findings	24 (7.1)	23 (16.9)	8 (17)	0.001*
Small intestine	2 (0.6)	2 (1.5)	0 (0)	0.600
Large intestine/rectum	16 (4.8)	15 (11.0)	6 (13)	0.001*
Carcinosis	6 (1.8)	6 (4.4)	2 (4)	0.100
**Method of operation**				
Laparotomy	92 (27.4)	28 (20.6)	12 (25)	0.120
Laparoscopy	123 (36.6)	19 (14.0)	10 (21)	<0.001*
Laparoscopy converted to laparotomy	121 (36.0)	89 (65.4)	26 (54)	<0.001*
**Resection**	138 (41.1)	71 (52.2)	20 (42)	0.020*
Stomach/duodenum	2 (1.4)	0 (0.0)	0 (0)	n.a.
Small bowel	78 (56.5)	35 (59.3)	5 (25)	0.560
Large bowel	58 (42.0)	36 (50.7)	15 (75)	0.020*
**Anastomosis**				
No	252 (75.0)	96 (70.6)	38 (79)	0.300
Yes	84 (25.0)	40 (29.4)	10 (21)	0.300
**Closure of abdominal wall**				
Primary suture	317 (94.3)	126 (92.6)	46 (96)	0.500
Intra-abdominal VAC treatment	19 (5.7)	9 (6.6)	2 (4)	0.500
Traction mesh and VAC	0 (0.0)	1 (0.7)	1 (2)	n.a.
**Mass closure**				
Yes	8 (2.4)	5 (1.5)	2 (4)	0.400

Values are *n* (%). *Statistically significant. †According to the surgical wound classification system: 1, clean; 2, clean contaminated; 3, contaminated; and 4, dirty. CFS, clinical frailty scale; SBO, small bowel obstruction; LBO, large bowel obstruction; VAC, vacuum-assisted closure.

### Postoperative complications

Patients in the CFS 1–3 group had significantly fewer total complications than patients in the CFS 4–6 group and patients in the CFS 7–9 group (120, 250, and 277 complications per 100 patients respectively, *P* < 0.001; *Table 3*). This was also demonstrated for organ-specific complications; postoperative pulmonary complications increased with increasing frailty (rates of 11.6%, 39.0%, and 52% for patients in the CFS 1–3, CFS 4–6, and CFS 7–9 groups respectively, *P* < 0.001). Pneumonia was the most frequent pulmonary complication (rates of 6.0%, 13.2%, and 19% for patients in the CFS 1–3, CFS 4–6, and CFS 7–9 groups respectively, *P* < 0.001). Cerebral complications were frequent (rates of 5.4%, 21.3%, and 17% for patients in the CFS 1–3, CFS 4–6, and CFS 7–9 groups respectively, *P* < 0.001) and delirium was the most likely cerebral complication to occur (rates of 2.7%, 18.4%, and 13% for patients in the CFS 1–3, CFS 4–6, and CFS 7–9 groups respectively, *P* < 0.001). Cardiac complications (rates of 7.1%, 20.6%, and 21% for patients in the CFS 1–3, CFS 4–6, and CFS 7–9 groups respectively, *P* < 0.001) and renal complications (rates of 5.4%, 18.4%, and 13% for patients in the CFS 1–3, CFS 4–6, and CFS 7–9 groups respectively, *P* < 0.001) also increased in the frail groups, with arrhythmia (rates of 3.6%, 8.8%, and 6% for patients in the CFS 1–3, CFS 4–6, and CFS 7–9 groups respectively, *P* < 0.001) and acute renal failure (rates of 3.3%, 11.0%, and 10% for patients in the CFS 1–3, CFS 4–6, and CFS 7–9 groups respectively, *P* < 0.001) being the most frequent cardiac and renal complications.

**Table 3:** Distribution of organ-specific postoperative complications according to the clinical frailty scale

	CFS 1–3, *n* = 336	CFS 4–6, *n* = 136	CFS 7–9, *n* = 48	*P*
Intrahospital complications per 100 patient, *n*	120	250	277	<0.001*
Thirty-day mortality	12 (3.6)	25 (18.4)	16 (33)	<0.001*
**Cardiac**	24 (7.1)	28 (20.6)	10 (21)	<0.001*
Cardiac arrest	8 (2.4)	11 (8.1)	4 (8)	0.004*
Arrhythmia (atrial fibrillation, atrial flutter)	12 (3.6)	12 (8.8)	3 (6)	0.020*
Other†	3 (0.9)	6 (4.4)	3 (6)	0.100
**Pulmonary**	39 (11.6)	53 (39.0)	25 (52)	<0.001*
Respiratory failure	7 (2.1)	10 (7.4)	3 (6)	0.005*
Pneumonia	20 (6.0)	18 (13.2)	9 (19)	0.009*
Pleural effusion	5 (1.5)	9 (6.6)	5 (10)	0.003*
Other‡	7 (2.1)	15 (11.0)	9 (19)	0.410
**Cerebral**	18 (5.4)	29 (21.3)	8 (17)	<0.001*
Delirium	9 (2.7)	25 (18.4)	6 (13)	<0.001*
Other§	9 (2.7)	5 (3.7)	2 (4)	0.180
**Renal**	18 (5.4)	25 (18.4)	6 (13)	<0.001*
Creatinine >27 μmol/l	11 (3.3)	15 (11.0)	5 (10)	<0.001*
Other¶	7 (2.1)	10 (7.4)	1 (2)	<0.001*
**Surgical**	108 (32.1)	56 (41.2)	32 (67)	0.060
Burst abdomen#	15 (5)	7 (5)	4 (8)	0.750
Anastomotic leakage**	5 (6)	0 (0)	1 (10)	n.a.
Intra-abdominal abscess	13 (3.9)	5 (3.7)	4 (8)	0.920
Intestinal paralysis (>7 days)	26 (7.7)	16 (11.8)	5 (10)	0.160
Intra-abdominal bleeding	6 (1.8)	5 (3.7)	2 (4)	n.a.
Other††	43 (12.8)	23 (16.9)	11 (23)	0.330
**Wound related**	36 (10.7)	16 (11.88)	3 (6)	0.740
Wound infection	14 (4.2)	7 (5.1)	1 (2)	0.640
Wound rupture	11 (3.3)	1 (0.7)	1 (2)	0.110
Other‡‡	11 (3.3)	8 (5.9)	1 (2)	0.210
**Infectious**	35 (10.4)	32 (23.5)	17 (35)	<0.001*
Sepsis	12 (3.6)	13 (9.6)	5 (10)	0.009*
Urinary tract infection	4 (1.2)	4 (2.9)	4 (8)	0.180
Other§§	19 (5.7)	15 (11.0)	8 (17)	0.070

Values are *n* (%) unless otherwise indicated. *Statistically significant. †Acute myocardial infarction, myocardical infarction after non-cardiac surgery, acute heart failure, worsening of heart failure, pulmonary embolism, deep-vein thrombosis. ‡Atelectasis, bronchial spasm, lung statis, pneumothorax. §Postoperative cognitive decline, depression, anxiety, intracranial haemorrhage, neuropathy. ¶Worsening of chronic kidney failure, dialysis, overhydration, electrolyte imbalance. #Number (%) of patients undergoing primary closure with suture. **Number (%) of patients undergoing primary anastomosis. ††Gastrointestinal tract bleeding, subileus, bowel obstruction, visceral ischaemia, reperforation. ‡‡Stoma separation, wound haematoma, incisional pain. §§Oral candida, unknown infectious focus, intestinal infection. CFS, clinical frailty scale.

No overall difference was found regarding surgical complications for patients in the CFS 1–3 group and for patients in the CFS 4–6 group (32.1% *versus* 41.2% respectively, *P* = 0.060), but patients in the CFS 7–9 group had more surgical complications compared with patients in the CFS 1–3 group (67% *versus* 32.1% respectively, *P* = 0.001). Differing results regarding rates of anastomotic leakage were seen, with 6% for patients in the CFS 1–3 group, 0% for patients in the CFS 4–6 group, and 10% for patients in the CFS 7–9 group (*P* = 0.400). No differences were found for burst abdomen or for wound-related infections when comparing non-frail patients with frail patients.

### Multivariate analysis

**Fig. 2:**
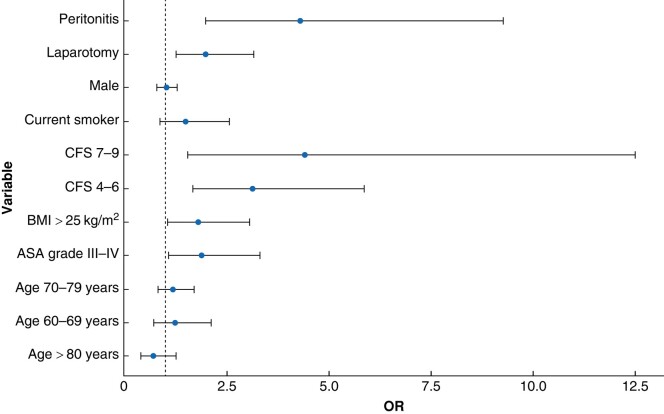
Multivariate regression analysis showing the risk of developing at least one postoperative complication during admission Peritonitis was defined according to the surgical wound classification system: 1, clean; 2, clean contaminated; 3, contaminated; and 4, dirty. CFS, clinical frailty scale.

In a multivariate analysis, variables associated with an increased risk of experiencing at least one postoperative complication were ASA grade III–IV (OR 1.90, 95% c.i. 1.10 to 3.30), peritonitis (OR 4.30, 95% c.i. 2.00 to 9.30), laparotomy (OR 2.00, 95% c.i. 1.30 to 3.10), CFS 4–6 (OR 3.10, 95% c.i. 1.70 to 5.80), CFS 7–9 (OR 4.40, 95% c.i. 1.60 to 12.50), and BMI greater than or equal to 25 (OR 1.80, 95% c.i. 1.10 to 3.10) (*Fig. 2*).

### Mortality

**Fig. 3:**
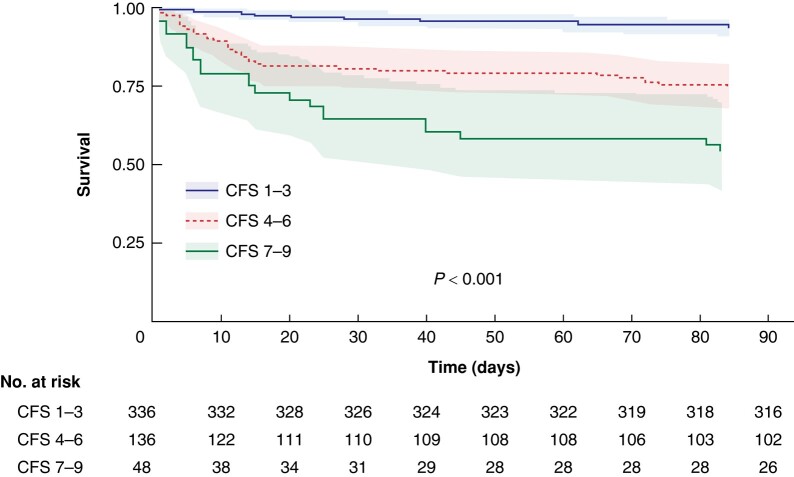
Survival curves and number at risk table showing the 90-day survival for each frailty group The shaded areas represent the 95% confidence intervals. CFS, clinical frailty scale.

There was a considerable difference in mortality rates for patients with differing levels of frailty, with patients in the CFS 1–3, CFS 4–6, and CFS 7–9 groups exhibiting mortality rates within 30 days of 3.6%, 18.3%, and 33.3% respectively (*P* < 0.001). The corresponding mortality rates within 3 days were 0.3%, 2.9%, and 10% respectively (*P* < 0.001), within 7 days were 0.9%, 8.8%, and 23% respectively (*P* < 0.001), and within 90 days were 7.6%, 26%, and 46% respectively (*P* < 0.001). The survival within 90 days is illustrated as a survival curve (*Fig. 3*) for each frailty group.

## Discussion

Patients with mild to moderate frailty (CFS 4–6) and severe frailty (CFS 7–9) were observed to have a significantly higher incidence of postoperative complications. A pronounced incidence of pulmonary complications, particularly pneumonia, was noted. Postoperative atrial fibrillation and delirium emerged as prevalent complications, particularly noteworthy in patients exhibiting clinical frailty. Additionally, patients with clinical frailty demonstrated an increased susceptibility to acute renal failure and postoperative sepsis as complications. Postoperative delirium and pulmonary complications are particularly well described in several studies, including studies with larger sample sizes, such as meta-analyses and systematic reviews^32–34^. Although the rate of laparoscopic surgery was notably high for patients in the CFS 1–3 group in this study, there is no indication that laparoscopic surgery is associated with the development of pulmonary complications. The incidence of pulmonary complications was significantly higher for patients in the CFS 4–6 and CFS 7–9 groups, who more frequently underwent laparotomy. The existing literature suggests that the increased rate of pulmonary complications after laparoscopy is more evident in individuals with preexisting pulmonary conditions and also that emergency surgery is an independent predictor of pulmonary complications^35,36^. No significant difference was detected in terms of surgical complications for patients with no frailty compared with patients with mild to moderate frailty. Patients with severe frailty had significantly more surgical complications than non-frail patients. Mortality within 3, 7, 30, and 90 days was markedly increased in frail patients compared with non-frail patients.

Frailty, a prevalent condition among older individuals, is linked to a greater likelihood of unfavourable outcomes after MEAS, such as an increased risk of postoperative complications^1,37–41^. The underlying mechanism for this connection is multifaceted, probably involving an impaired healing process, immunosenescence, and reduced functional capacity^42^. However, most of the research in this area has focused on the elective setting, where patients undergo planned surgery after an interval of preparation^39,43–45^. Frailty is an independent predictor of postoperative mortality in various surgical populations, including patients undergoing elective major abdominal surgery, cardiac surgery, and orthopaedic surgery^14,15,46^. In the emergency setting, there is some evidence supporting the findings in this study, including an increased overall rate of postoperative complications, especially short-term mortality^47–51^. These studies propose various factors contributing to this phenomenon, including limited time for preoperative optimization, heightened physiological stress, and constrained psychological resources in emergency settings.

The limitations of this study include that this study cannot definitively determine whether the observed connections between frailty and postoperative complications result from the presence of other health conditions in frail patients, the inherent nature of frailty as a stand-alone predictor of postoperative issues, or a combination of both factors. Furthermore, the risk of bias is an inherent limitation of a single-centre cohort and a short observational interval (<2 years). However, given the prospective design and the relatively large sample size in a large emergency facility, the data are believed to reflect a general tendency in patients undergoing MEAS.

This study highlights the importance of identifying and addressing frailty in the emergency department and might indicate the need for preoperative targeting and risk profiling of patients undergoing MEAS. This could potentially reduce the risk of intrahospital postoperative complications and improve overall quality of life for this patient group. Finally, this study underscores the importance of interdisciplinary collaboration between emergency medicine, surgical teams, and geriatric specialists. Further research is needed to explore interventions and strategies that can mitigate the risks associated with frailty in this population.

This study demonstrates that frailty significantly associates with a specific postoperative complication pattern after MEAS. Patients with frailty have a higher incidence of complications—notably medical complications—and increased mortality rates compared with non-frail patients.

## Data Availability

The data that support the findings in this article are available on request from the corresponding author.
